# PTN/IGF-2 signaling modulates endometrial decidualization and immune cell trafficking to facilitate pregnancy maintenance

**DOI:** 10.3389/fimmu.2026.1790942

**Published:** 2026-03-23

**Authors:** Dezhao Chen, Quanrong Li, Huili Yang, Jiawei Shi, Peifang Chen, Tongfei Wang, Ling Zhou, Weichao Dai, Luo Zheng, Mingqing Li, Jie Zhang, Zhenzhen Lai

**Affiliations:** 1 Department of Obstetrics and Gynecology, Fujian Medical University Union Hospital, Fuzhou, China; 2 Department of Reproductive Immunology, The International Peace Maternity and Child Health Hospital, School of Medicine, Shanghai Jiao Tong University, Shanghai, China; 3 Department of Obstetrics and Gynecology, The First Affliated Hospital of Ningbo University, Ningbo, China; 4 Department of Gynecology, Obstetrics and Gynecology Hospital, Fudan University, Shanghai, China

**Keywords:** decidualization, IGF-2, immune regulation, PTN, recurrent implantation failure, recurrent pregnancy loss

## Abstract

**Background:**

Recurrent implantation failure (RIF) and recurrent pregnancy loss (RPL) are major causes of pathological early pregnancy, yet their mechanisms remain poorly understood. This study aimed to identify shared molecular mediators and their roles in endometrial dysfunction and immune regulation.

**Methods:**

Gene expression datasets for RIF and RPL were analyzed for differentially expressed genes (DEGs), functional enrichment, and protein–protein interaction (PPI) networks. Key regulators were identified using CytoHubba and Random Forest, and receiver operating characteristic (ROC) analysis evaluated their diagnostic performance. Endometrial stromal cells (ESCs) from RIF or RPL patients were used for *in vitro* functional assays, and *in vivo* murine models with *in situ* uterine PTN knockdown and IGF-2 rescue were established to assess pregnancy outcomes. RT-qPCR, immunoblotting, immunofluorescence, and flow cytometry were performed to assess decidualization markers and immune cell compositions.

**Results:**

PPI network and machine learning analysis identified PTN as a central hub gene shared by RIF and RPL. ROC curves showed that PTN had the highest diagnostic value among all candidate genes. Immunofluorescence confirmed that PTN is mainly expressed in ESCs and downregulated in RIF and RPL patients. *In vitro*, PTN promoted decidualization markers (IGFBP1, PRL, IGF-2, WNT4, and LIF) and modulated immune cell composition via IGF-2. *In vivo*, uterine PTN knockdown impaired implantation, reduced embryo numbers, and increased embryo resorption rates, while IGF-2 supplementation partially rescued these defects. These results indicate that PTN regulates ESC decidualization and endometrial immune tolerance and serves as a highly predictive biomarker for pathological pregnancies in RIF and RPL.

**Discussion:**

The PTN/IGF-2 axis promotes ESC decidualization and a tolerogenic immune microenvironment, supporting endometrial receptivity. Dysregulation of this pathway may underlie pathological pregnancies (including RIF and RPL), highlighting PTN as a potential therapeutic target for early pregnancy loss.

## Graphical Abstract

**Figure:**
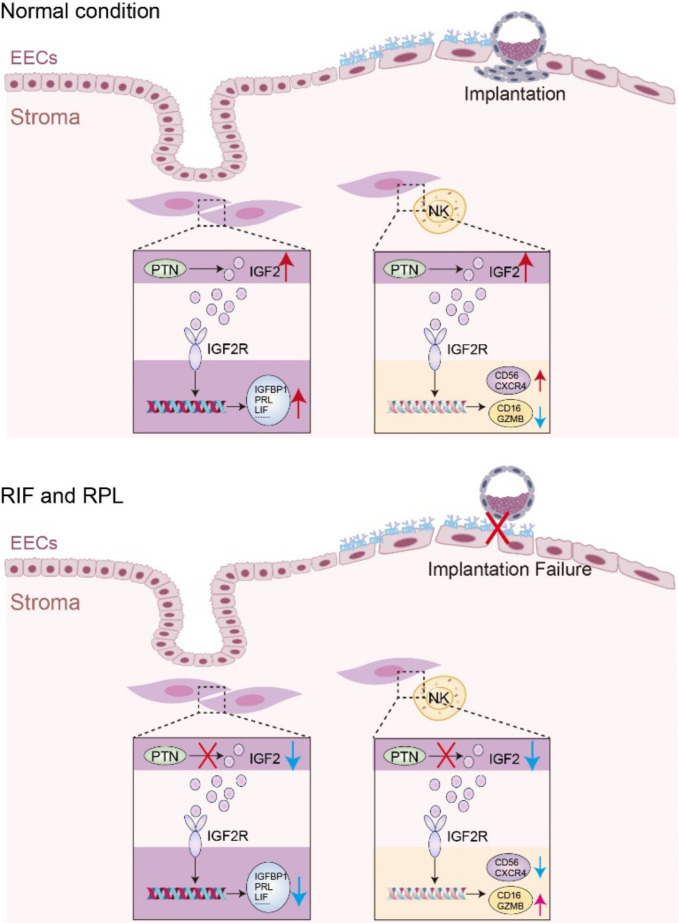

## Highlights

- During the implantation window, ESCs exhibited elevated PTN expression, which in turn activated downstream IGF-2 signaling pathways and facilitated decidualization.
- PTN/IGF-2 produced by ESCs facilitated the recruitment and redistribution of local immune effector cells, particularly NK cells, within the endometrial microenvironment.
- During the peri-pregnancy phase, the downregulation of PTN expression in ESCs contributed to decidualization defects and dysregulated immune cell trafficking, ultimately causing recurrent early pregnancy loss (RIF and RPL).

## 1 Introduction

Reproductive failures, characterized by the inability to achieve or maintain a successful pregnancy, such as recurrent implantation failure (RIF) and recurrent pregnancy loss (RPL), are two major clinical challenges in reproductive medicine that severely affect patients’ reproductive outcomes and psychological well-being in approximately 10%–15% of women undergoing assisted reproductive technology (ART) and 1%–2% of women of reproductive age, respectively (1, 2). Although they are clinically distinct—RIF is typically defined as the unsuccessful implantation of three or more high-quality embryos or at least two embryo transfers from egg donation (3, 4), whereas RPL refers to the loss of two or more consecutive pregnancies before 20–24 weeks of gestation, affecting approximately 3%–5% of women attempting conception (5, 6)—emerging evidence suggests that RIF and RPL may share underlying endometrial and immunological abnormalities that contribute to impaired embryo implantation and early pregnancy loss (7).

Most existing studies have focused on the individual etiologies of RIF and RPL, including factors such as embryo quality, endometrial receptivity, immune dysregulation, coagulation defects, and structural or chromosomal abnormalities (e.g., uterine anomalies, hydrosalpinx, or parental karyotype anomalies) (8, 9). However, few have systematically investigated the potential shared mechanisms between these conditions. Recent studies have suggested that RIF and RPL patients may exhibit overlapping features in immune marker expression, gene profiles, and signaling pathways. For instance, both conditions have been associated with altered uterine NK cell activity, reduced M2 macrophages and myeloid-derived suppressor cells, an increased Th1/Th2 ratio, a decreased Treg/Th17 ratio, shared Human Leukocyte Antigen (HLA) alleles between partners, and a predisposition to autoimmune disorders (7).

Despite these observations, comprehensive multi-omics analyses specifically addressing the comorbidity between RIF and RPL are still lacking. Elucidating their shared molecular mechanisms could facilitate the identification of common diagnostic biomarkers and therapeutic targets (10), enabling more precise clinical classification and the development of individualized treatment strategies for patients facing reproductive failure (11).

In this study, we aimed to investigate the shared molecular mechanisms underlying RIF and RPL. By integrating transcriptomic data from both conditions and focusing on key regulatory pathways, we sought to identify candidate molecules that may coordinate endometrial function and immune regulation. Our work provides a framework for understanding the molecular basis of early pregnancy failure and highlights potential targets for therapeutic intervention.

## 2 Materials and methods

### 2.1 RIF and RPL datasets

Four independent RIF gene expression profiles (GSE188409, GSE111974, GSE58144, and GSE183837) and three independent RPL gene expression profiles (GSE165004, GSE26787, and GSE71835) were downloaded from the Gene Expression Omnibus (GEO) database (https://www.ncbi.nlm.nih.gov/geo/). GSE188409 and GSE111974 were exploited as discovery datasets to identify differentially expressed genes (DEGs) of RIF; GSE165004 and GSE26787 were exploited as discovery datasets to identify DEGs of RPL. GSE58144 and GSE183837 were selected as the validation datasets of RIF. GSE71835 was selected as the validation dataset of RPL. GSE188409 is an expression profile based on the GPL26963 platform (Agilent-085982 Arraystar human lncRNA V5 microarray) and contains samples of fertile control (n = 5) and RIF (n = 5). GSE111974 contained gene expression profiles of 48 endometrium samples (24 control endometrium samples and 24 RIF endometrium samples). GSE188409 included 10 endometrium samples (five control endometrium samples and five RIF endometrium samples). GSE58144 contained 71 control endometrium samples and 43 RIF endometrium samples. GSE183837 was a single-cell RNA-seq (scRNA-seq) dataset of fertile control (n = 3) and RIF (n = 6). The GSE165004 database contained gene expression profiles of 48 endometrium samples (24 control endometrium samples and 24 RPL endometrium samples). GSE26787 included 10 endometrium samples (five control endometrium samples and five RPL endometrium samples). GSE71835 contained six control endometrium samples and six RPL endometrium samples. The inclusion and exclusion criteria of this study are listed in **Table 1**.

**Table 1:** Baseline characteristics.

	RIF				RPL		
	Training data		Test data		Training data		Test data
	GSE111974	GSE188409	GSE58144	GSE183837	GSE165004	GSE26787	GSE71835
Techniques	Agilent-039494 SurePrint G3 Human GE v2 8x60K Microarray 039381	GPL26963 platform	GPL15789 platform	10X Genomics Chromium platform	GPL16699 platform	GPL570 platform	GPL10558 platform
Sample size	48 (24 control samples and 24 RIF samples)	10 (5 control samples and 5 RIF samples)	114 (71 control samples and 43 RIF samples)	9 (3 control samples and 6 RIF samples)	48 (24 control samples and 24 RPL samples)	10 (5 control samples and 5 RPL samples)	12 (6 control samples and 6 RPL samples)
Inclusion criteria	Ctrl: had a history of at least one live birth. RIF: had a history of implantation failure from at least three consecutive In Vitro Fertilization (IVF) attempts (including a total of ≥4 good-quality embryos).				Ctrl: regularly cycling women aged under 35 years with at least one live birth. RPL: regularly cycling women aged under 35 years with at least two consecutive pregnancy losses of 20 weeks or less.		
Exclusion criteria	1. Tubal obstruction (tubal obstruction factor on hydrosalpinx, salpingitis, etc., was excluded). 2. Active pelvic infections, undiagnosed vaginal bleeding, uterine anomalies, endometriosis, and karyotype anomalies in one or both partners. 3. Unexplained infertility.				1. Associated gynecologic (endometriosis, fibroids, and active or history of pelvic inflammatory disease) or other medical comorbidities (hyperprolactinemia, thyroid disease, etc.). 2. Active pelvic infections, undiagnosed vaginal bleeding, uterine anomalies, endometriosis, and karyotype anomalies in one or both partners.		

RIF, recurrent implantation failure; RPL, recurrent pregnancy loss.

### 2.2 Data preprocessing

The “limma” package (12) in the R software (version 4.2.0; www.r-project.org was used for the background correction and quantile normalization of all the raw data files, and the expression values were then obtained. The averages of the probe set of values were calculated as the expression values for the same gene with multiple probe sets (13).

### 2.3 Identification of DEGs

To establish the DEGs of RIF and RPL, the “limma” package of R (12) was used. Moreover, a volcano plot was generated to assess the DEGs. Genes with | log2 fold change (FC) | ≥ 0.58 and adjusted p-value < 0.05 were taken as differentially expressed genes between RIF and control endometrium. A volcano map of DEGs was drawn using the GraphPad Prism v10.0 software.

### 2.4 Functional enrichment of DEGs

The Gene Ontology (GO) analysis and Kyoto Encyclopedia of Genes and Genomes (KEGG) pathway analysis of co-expressed genes were carried out using the R software and the “clusterProfiler” package (14). In this analysis, symbol codes were first converted to Entrez ID using the human genome annotation package “org.Hs.eg.db”, and the “ggplot2” (15), “pathview” (16), and “gplots” packages of the R software were used to visualize the plots.

### 2.5 Protein–protein interaction network analysis and hub gene extraction

The STRING database (available online: http://string-db.org) was used for protein–protein interaction (PPI) network prediction. Cytoscape (v. 3.8.1) was used for visual representation and further PPI network experimental studies. Hub genes play an important role in biological processes. Based on the PPI network, hub genes were screened according to network topology. The Cytoscape software (version 3.8.1, CytoHubba and MCODE plug-ins) was used to discover the key targets or subnetworks of complex networks (17, 18).

### 2.6 Random Forest analysis

The Random Forest model has gained popularity as a tree-based ensemble learning algorithm that is utilized extensively in the field of medicine. This model has proven to be of high accuracy and is considered user-friendly. One of its key features is its integrated feature selector that automatically identifies the significance of various attributes throughout the training phase. The mean decrease Gini is a forest-wide weighted average that measures the decrease in the Gini impurity. Using the Random Forest algorithm, we could compute the importance value of common DEGs and subsequently identify the top 20 genes based on their feature importance.

### 2.7 Identification of overlapped hub genes

After identifying hub genes using the Cytoscape software or Random Forest analysis of RIF and RPL separately, they were imported into the R software to obtain their intersection. These jointly dysregulated genes may be the keys to the links between the two, and we call them overlapped hub genes.

### 2.8 Immune infiltration analysis

The different immune cell types of tissues were analyzed by CIBERSORT (19) using normalized gene expression profiles of GSE188409 and GSE111974 for RIF, and GSE165004 and GSE26787 for RPL. Next, a matrix of 22 kinds of immune cells was obtained. CIBERSORT p < 0.05 was used to filter the samples, and the percentage of each immune cell type in the samples was calculated and displayed in a bar plot. The “box” package was used to compare the levels of 22 kinds of immune cells between the two groups. The “*ggplot2*” package (15) was adopted to visualize the plots.

### 2.9 ROC analysis

The multivariate modeling with combined selected overlapped hub genes was used to identify biomarkers with high sensitivity and specificity for RIF and RPL diagnoses using a visualization tool (https://hiplot.com.cn/basic/roc). The GSE188409, GSE111974, GSE165004, and GSE26787 datasets as training samples and the GSE58144 and GSE71835 datasets as validation samples were used iteratively. The receiver operating characteristic curves were plotted, and the area under the curve (AUC) was calculated separately to evaluate the performance of each model using the R package “*pROC*” (20). AUC > 0.7 indicated that the model had a good fitting effect.

### 2.10 Patients for the cohort of validation

The protocol for this study was approved by the Human Research Ethics Committees of Fujian Medical University Union Hospital, and written informed consent was obtained from all participants. Human endometrial tissues were collected from women attending the Department of Assisted Reproduction, a dedicated research clinic at the Fujian Medical University Union Hospital. Surplus tissue from endometrial biopsies obtained for diagnostic purposes at the Department of Assisted Reproduction was used for this study. The timing of endometrial biopsy was 5 days after ovulation [ultrasonic observation, equate to Luteinizing Hormone (LH)+7, the window of implantation (WOI) time] in a natural cycle. The definition of RIF and RPL was consistent with the studies recruited for bioinformatics. The control group comprised women who had a history of pregnancy but received assisted reproductive technology due to obstruction of the fallopian tube or male infertility. All the samples were transported to the laboratory on ice in Dulbecco’s modified Eagle’s medium (DMEM)/F-12 (Gibco) for further study.

### 2.11 Sample preparation

The procedures of sample preparation and endometrial stromal cell (ESC) isolation were described in detail in our previous studies (21). In brief, the endometrial tissue samples were minced into 1-mm^3^ pieces on ice and subsequently digested with 1 mg/mL collagenase type IV (Sigma-Aldrich, St. Louis, MO, USA, 9001-12-1). The tissue pieces were later filtered through a 70-μm cell strainer (Falcon, 431752), followed by centrifugation at 400 × *g* for 8 min for the collection of all types of cells. Later, the cells were resuspended in DMEM/F-12 containing 10% fetal bovine serum (FBS; HyClone, Logan, UT, USA), plated on culture flasks, and incubated in a humidified incubator with 5% CO_2_ at 37 °C. Finally, after 24 h, the supernatant was removed and centrifuged at 400 × *g* at 4 °C for flow cytometry analysis. The remaining cells were digested with trypsin and inoculated into a new culture dish, and they were treated with control vehicle or IGF-2 (50 ng/mL, R&D Systems, Minneapolis, MN, USA, 292-G2) for 48 h and then collected for quantitative real-time polymerase chain reaction (qRT-PCR).

### 2.12 Cell line culture, treatment, and cell transfection

Human endometrial stromal cells (hESCs; CRL-4003) were cultured in DMEM/F-12 medium supplemented with 10% FBS from HyClone (GE Healthcare, Chicago, IL, USA, SH30088.03), along with 100 U/mL penicillin and 100 μg/mL streptomycin sourced from Gibco (Thermo Fisher Scientific, MA, USA, 15140122). These cells were plated in culture flasks and incubated in a humidified environment containing 5% CO_2_ at 37 °C.

To silence the *PTN* gene in hESCs, recombinant lentivirus of siRNA targeting *PTN* (Genechem, Shanghai, China) or control lentivirus with GFP from Shanghai Genechem Co., Ltd. (China) was used to infect hESCs for 6 h. Six hours after infection, fresh complete culture medium was used to continue the culture. All transfection experiments applied Lipofectamine™ 3000 reagents (Thermo Fisher Scientific, L3000015) according to the manufacturer’s protocols. The infection efficiency was evaluated via Western blotting 48 h after infection. Then, these cells were treated with control vehicle, cyclic adenosine monophosphate (cAMP; 0.5 mM, MCE, HY-12306), or IGF-2 (50 ng/mL), measuring the expression levels of *PTN*, *IGF-2*, *PRL*, *IGFBP1*, *WNT4*, and *LIF* via RT-qPCR.

### 2.13 SiRNA-mediated knockdown of *Ptn* in mouse endometrium

For siRNA-mediated knockdown of *Ptn*, the lumen of the right uterine horn was injected with 50 μL of a mixture of Lipofectamine RNA iMAX reagent (Invitrogen, Carlsbad, CA, USA, 13778075) and 60 pmol of either non-targeting siRNA or siRNAs targeting *Ptn* (Genechem, Shanghai, China). Forty-eight hours later, mice were injected with IGF-2 (50 ng/mL) through the tail vein for 48 h. Then, a part of the uterus was collected and fixed in 4% paraformaldehyde for subsequent sectioning, or used to measure mRNA expression levels via RT-qPCR, or digested with 5 mg/mL collagenase type IV, and cell suspension was collected to measure the proportions of immune cells via flow cytometry analysis (FCM). The remaining mice were mated with male mice (2:1) to construct pregnant mouse models (vaginal plug = day 0.5 of pregnancy). On the 13.5th day of pregnancy, the number of embryos implanted was recorded.

### 2.14 Flow cytometry analysis

FCM was used to identify different subsets of immunocytes. All antibodies were from BioLegend (San Diego, CA, USA). The antibodies used were the following: APC/Cyanine 7 (APC/Cy7)-conjugated anti-human CD45, fluorescein isothiocyanate (FITC)-conjugated anti-human CD3, brilliant violet (BV) 510-conjugated anti-human CD56, PE-Cy7-conjugated anti-human CD4, APC-conjugated anti-human CD8, and PE-conjugated anti-human CD16. APC/Cy7-conjugated anti-mouse Cd45, FITC-conjugated anti-mouse Cd3, BV 510-conjugated anti-mouse NK1.1, PE-Cy7-conjugated anti-mouse Cd4, APC-conjugated anti-mouse Cd8, PE-conjugated anti-mouse Cd16, BV 421-conjugated anti-mouse GZMB, and PE-conjugated anti-mouse Cxcr4. Staining was performed using the above antibodies (5 μL separately) at room temperature for 30 min in the dark. In addition, an isotype IgG antibody (5 μL separately) was used as the control. Human TruStain FcX (cat. no. 422301; BioLegend, Inc.) was used to block Fc receptors prior to flow cytometry. Subsequently, cells were washed twice and resuspended in Phosphate-Buffered Saline (PBS) for flow cytometry analysis. Samples were analyzed using a CytoFLEX flow cytometer (Beckman Coulter, Inc., Brea, CA, USA), and data were analyzed using FlowJo (version 10.07, FlowJo LLC, Ashland, OR, USA)).

### 2.15 Western blotting

The proteins were isolated using Radioimmunoprecipitation assay (RIPA) buffer, separated by sodium dodecyl sulfate–polyacrylamide gel electrophoresis (SDS-PAGE), and subsequently transferred to polyvinylidene fluoride (PVDF) membranes. The cells were treated with a blocking buffer (5% bovine serum albumin (BSA)) at room temperature for 1 h and then exposed to antibodies against PTN (1:1,000; Cell Signaling Technology, Danvers, MA, USA, 54934S), IGFBP1 (1:1,000; Cell Signaling Technology, Danvers, MA, USA, 64143S), PRL (1:1,000; Cell Signaling Technology, Danvers, MA, USA, 79418S), and GAPDH (1:1,000; Cell Signaling Technology, Danvers, MA, USA, 14C10) at 4 °C overnight. Then, the PVDF membranes were exposed at room temperature for 1 h to peroxidase-conjugated goat anti-rabbit IgG secondary antibodies (1:5,000; Bioworld Technology, Co. Ltd., Dublin, OH, USA, BS20241-Y). Subsequently, the membrane was washed three times and subjected to chemiluminescence using the Immobilon Western Chemiluminescent HRP Substrate Kit (Millipore, Burlington, MA, USA).

### 2.16 Immunofluorescence

Endometrial tissue sections were fixed, blocked, and incubated with the following antibodies at 4 °C overnight: PTN (1:1,000; Cell Signaling Technology, Danvers, MA, USA, 54934S) and Vimentin (1:1,000; Abcam, Cambridge, MA, USA, ab8978). The secondary antibodies used and dilution ratios were as follows: donkey anti-rabbit (1:500, Abcam, Cambridge, MA, USA, ab150075) and goat anti-mouse (1:500, Abcam, Cambridge, MA, USA, ab150117). All sections were counterstained with DAPI (D1306, Thermo Fisher Scientific) and mounted in buffered glycerol. They were imaged using an optical and epifluorescence microscope (BX53 microscope,Olympus, Tokyo, Japan).

### 2.17 Enzyme-linked immunosorbent assay

IGF-2 levels in cell culture supernatants were measured using a commercial human IGF-2 enzyme-linked immunosorbent assay (ELISA) kit (Boster, Wuhan, China) according to the manufacturer’s instructions. Briefly, culture supernatants were collected at the indicated time points, centrifuged at 1,000–2,000 × *g* for 10 min at 4 °C to remove debris, and stored at −80°C until analysis. After equilibration to room temperature, standards and appropriately diluted samples were added to antibody-precoated 96-well plates and incubated as instructed, followed by sequential incubation with biotin-conjugated detection antibody and horseradish peroxidase (HRP)-conjugated streptavidin. After 3,3′,5,5′-tetramethylbenzidine (TMB) substrate development and reaction termination, absorbance was read at 450 nm. IGF-2 concentrations were calculated from the standard curve and normalized to cell number or total protein, where indicated.

### 2.18 Quantitative real-time polymerase chain reaction

The total RNA was extracted using TRIzol reagent (Invitrogen, 15596026CN). Subsequently, the concentration and purity of RNA were quantified using a NanoDrop spectrophotometer (NanoDrop Technologies; Thermo Fisher Scientific, MA, USA). The PrimeScript RT Reagent Kit (Takara Bio Inc., Kusatsu, Shiga, Japan) was utilized to reverse-transcribe total RNA to cDNA. Next, qRT-qPCR was performed using SYBR Green PCR Master Mix (TaKaRa Biotechnology, Kusatsu, Shiga, Japan, 639676). The qRT-PCR primers are listed in **Table 2**. The target mRNA expressions were normalized to ACTB expression. All reactions were processed on the Applied Biosystems 7500 Real-Time PCR System (Thermo Fisher Scientific, MA, USA). The test results were analyzed using the 2^−ΔΔCt^ method.

**Table 2:** Gene primers for qRT-PCR.

Gene name	Forward primer (5′–3′)	Reverse primer (5′–3′)
*Gapdh*	AAGAAGGTGGTGAAGCAGGCATC	CGGCATCGAAGGTGGAAGAGTG
*Igfbp1*	CCGCCACGAGCACCTTGTTC	AGCTGCTCCTCTGTCATCTCTGG
*Igf2*	GTGCTGCATCGCTGCTTAC	ACGTCCCTCTCGGACTTGG
*Lif*	GCCTCCAGGTCAAGCTCAATGC	ACACGGTACTTGTTGCACAGACG
*Ptn*	ATGTCGTCCCAGCAATATCAGC	CCAAGATGAAAATCAATGCCAGG
*Wnt4*	AGACGTGCGAGAAACTCAAAG	GGAACTGGTATTGGCACTCCT
*Prl*	CAGGGGTCAGCCCAGAAAG	TCACCAGCGGAACAGATTGG
*Il15*	AGAGGCCAACTGGATAGATGT	AGAGCACGTTTCTTACTGTTTCA
*Cxcr4*	CCAAGTGCTGCCGTCATTTTC	GGCTCGCAGGGATGATTTCAA
*GAPDH*	TGGTGAAGGTCGGTGTGAAC	GCTCCTGGAAGATGGTGATGG
*IGFBP1*	CGAAGGCTCTCCATGTCACCA	TGTCTCCTGTGCCTTGGCTAAAC
*IGF-2*	GTGGCATCGTTGAGGAGTG	CACGTCCCTCTCGGACTTG
*LIF*	CCAACGTGACGGACTTCCC	TACACGACTATGCGGTACAGC
*PRL*	AAGCTGTAGAGATTGAGGAGCAAAC	TCAGGATGAACCTGGCTGACTA
*PTN*	GGAGCTGAGTGCAAGCAAAC	CTCGCTTCAGACTTCCAGTTC

### 2.19 Statistical analysis

Statistical analysis was performed using the SPSS (version 28.0, Chicago, IL, USA) and GraphPad Prism (version 10.6, San Diego, CA, USA) software. Each experiment was independently conducted at least three times. Student’s t-test was employed for data with only two groups, while for data with more than two groups, a one-way analysis of variance (ANOVA) test was used, followed by Tukey’s or Bonferroni’s test for t-tests and χ^2^ test. The results were reported as mean ± SEM. The sensitivity and specificity of feature genes to distinguish RIF from controls were assessed using a receiver operating characteristic (ROC) curve. Differences were considered statistically significant when p < 0.05.

## 3 Results

### 3.1 The co-expressed DEGs and enriched pathways in RIF and RPL

**Figure 1:**
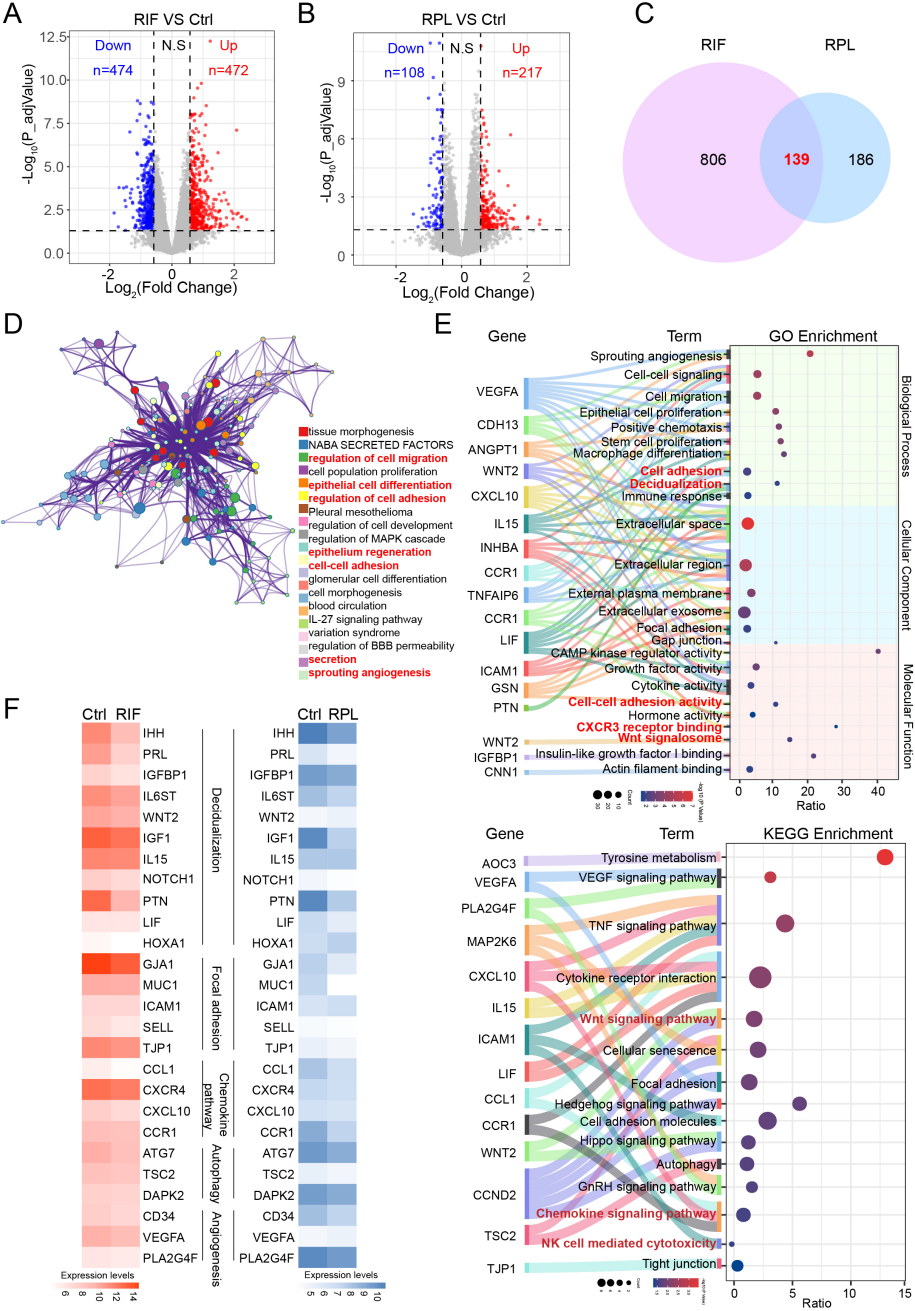
The co-expressed DEGs and enriched pathways in RIF and RPL. **(A)** Expression of DEGs in RIF and Ctrl groups is presented by volcano plot. **(B)** Expression of DEGs in RPL and Ctrl groups is presented by volcano plot. **(C)** Venn diagram showing the co-expressed DEGs between RIF and RPL groups. **(D)** Pathway enrichment analysis of the co-expressed DEGs using Metascape. **(E)** GO and KEGG enrichment analyses of the co-expressed DEGs. **(F)** Heatmap showed relative expression levels of selected genes in datasets of the RIF database (datasets GSE188409 and GSE111974) and the RPL database (datasets GSE165004 and GSE26787). These datasets were also used for the volcano plot analyses shown in panels A and **(B)** DEGs, differentially expressed genes; RIF, recurrent implantation failure; RPL, recurrent pregnancy loss; GO, Gene Ontology; KEGG, Kyoto Encyclopedia of Genes and Genomes.

Expression data from two RIF-related datasets (GSE111974 and GSE188409) were normalized prior to analysis. Volcano plots illustrating DEGs in the RIF datasets are shown in **Figures 1A**, which includes 474 downregulated genes and 472 upregulated genes. Moreover, the heatmap depicting the top 50 DEGs between the RIF and control groups is presented in **Supplementary Figures 1A**, and principal component analysis (PCA) was performed to visualize data distribution before and after normalization, as shown in **Supplementary Figure 1B**. Similarly, volcano plots, heatmaps, and PCAs of the RPL datasets (GSE165004 and GSE26787) are displayed in **Figure 1B**, **Supplementary Figures 1C, D**, respectively, revealing a total of 325 DEGs, in which 217 were upregulated and 108 were downregulated. To explore potential shared mechanisms between RIF and RPL, 139 overlapping DEGs were identified and used to construct a shared regulatory network (**Figure 1C**). Functional enrichment analysis of the shared DEGs using Metascape revealed significant enrichment in pathways related to cell migration, differentiation, adhesion, secretion, and angiogenesis (**Figure 1D**). GO enrichment analysis further demonstrated that the overlapping genes are primarily involved in cell adhesion processes, decidualization, CXCR3 receptor binding, and Wnt signaling (**Figure 1E**). Kyoto Encyclopedia of Genes and Genomes (KEGG) pathway analysis indicated their participation in TNF signaling, cytokine receptor interactions, Wnt signaling, chemokine signaling pathway, and NK cell-mediated cytotoxicity (**Figure 1E**). Since the pathways of decidualization, focal adhesion, chemokine pathway, autophagy, and angiogenesis play a crucial role in successful pregnancy, we found that the important genes of these pathways were decreased in both RIF and RPL patients (**Figure 1F**).

### 3.2 Identification of PTN as a shared hub gene with high diagnostic performance in RIF and RPL

**Figure 2:**
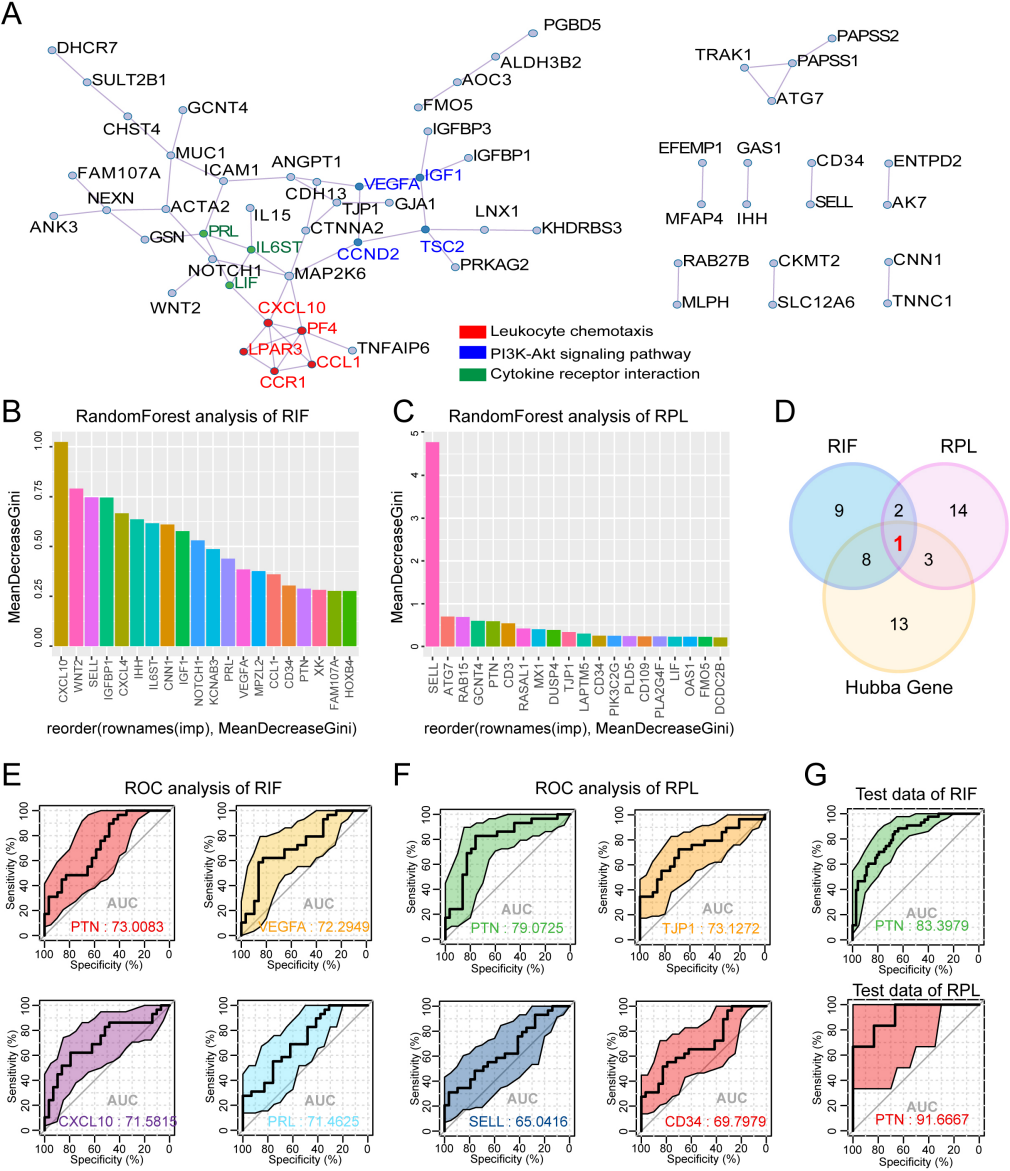
Identification of PTN as a shared hub gene with high diagnostic performance in RIF and RPL. **(A)** PPI network constructed with the co-expressed DEGs in both RIF and RPL. MCODE was used to identify the significant module from the PPI network with a score of ≥5.0. Different node colors represent different functions. **(B, C)** The mean decrease Gini score rank of the top 20 hub genes in RIF **(B)** and RPL **(C)** was calculated using Random Forest analysis. **(D)** Venn diagram showing the common hub genes calculated using Random Forest analysis of RIF and RPL, and Cytoscape analysis (**Supplementary Figure 2**). **(E)** ROC analysis confirmed the diagnostic efficacy of the top four biomarkers (the common hub genes identified between RIF and Hubba Gene) for RIF in training datasets. **(F)** ROC analysis confirmed the diagnostic efficacy of the four biomarkers (the common hub genes identified between RPL and Hubba Gene) for RPL in training datasets. **(G)** ROC analysis of the diagnostic efficacy of PTN for RIF (test data: GSE58144) and RPL (test data: GSE71835) in validation datasets. RIF, recurrent implantation failure; RPL, recurrent pregnancy loss; PPI, protein–protein interaction; DEGs, differentially expressed genes; ROC, receiver operating characteristic.

In order to identify shared hub genes in RIF and RPL, the PPI network of overlapped DEGs in RIF and RPL was first analyzed, and three key functional modules were identified using MCODE components that were extracted, predominantly associated with leukocyte chemotaxis, the PI3K–Akt signaling pathway, and cytokine–receptor interactions (**Figure 2A**). To further assess the significance of genes within the PPI network, the top 10 hub genes were identified using CytoHubba based on 10 different algorithms (**Supplementary Figure 2A**). The frequency of occurrence of each gene across these algorithms is presented in **Supplementary Figures 2B**, highlighting MAP2K6, PRL, PF4, and CXCL10 as potential key regulators. In addition, Random Forest analysis was employed to rank the top 20 DEGs based on their relative importance in RIF and RPL (**Figures 2B, C**). Next, by intersecting the top 20 most important genes from the Random Forest analysis with the hub genes identified using CytoHubba, a Venn diagram was constructed (**Figure 2D**), revealing a single overlapping gene, PTN, which was selected for subsequent validation. To further refine the candidate hub genes, ROC curve analysis was performed to evaluate the diagnostic performance of the top four genes with the highest AUC values in the training dataset of RIF and RPL, among which PTN achieved the highest AUC of 0.73 for RIF. Similarly, in the RPL group, PTN demonstrated the highest AUC of 0.79 (**Figures 2E, F**). Further validation using an independent dataset (GSE58144 for RIF and GSE71835 for RPL) confirmed the strong diagnostic potential of PTN, with an AUC > 0.8 in both conditions (**Figure 2G**). Taken together, these findings suggest that PTN may serve as a critical biomarker involved in the pathophysiology of both RIF and RPL.

### 3.3 Downregulation of PTN in pathological pregnancy conditions, including RIF and RPL

**Figure 3:**
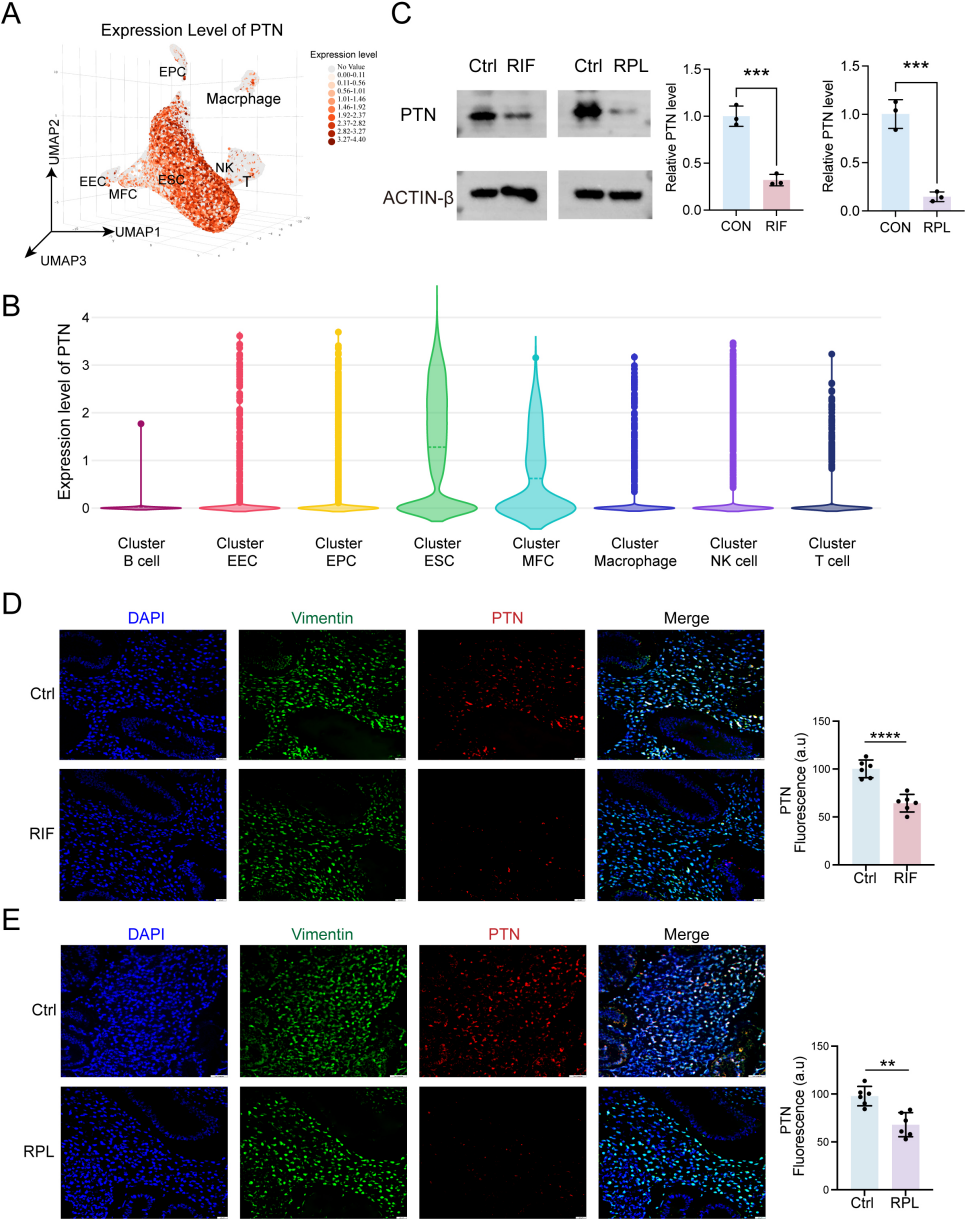
Downregulation of PTN in pathological pregnancy conditions, including RIF and RPL. **(A)** Uniform Manifold Approximation and Projection (UMAP) showing expression of PTN in RIF single-cell data from GSE183837. **(B)** Expression of PTN in different cell clusters in violin plots. **(C)** Expression levels of PTN in hESCs isolated from endometrium of RIF and RPL patients via Western blotting, which followed densitometric analysis of Western blotting band (n = 3). **(D, E)** Expression of PTN and Vimentin in RIF **(D)** and RPL **(E)** clinical samples by immunofluorescence, and quantitative analysis of immunofluorescence was performed (n = 6). Data are presented as mean ± SEM and analyzed using t-test (*p < 0.05, **p < 0.01, ****p < 0.0001). RIF, recurrent implantation failure; RPL, recurrent pregnancy loss; hESCs, human endometrial stromal cells.

In order to investigate the expression profile and potential functional role of PTN in RIF and RPL, we first analyzed its expression in single-cell transcriptomic data (GSE183837). As illustrated in **Figure 3A, B**, PTN was predominantly expressed in ESCs. Consistent with these findings, Western blotting analysis of ESCs isolated from patients further confirmed reduced PTN protein levels in both conditions (**Figure 3C**). We further validated its expression by co-staining PTN and Vimentin (a marker of ESCs) in clinical endometrial samples, followed by quantitative analysis. The results revealed a significant downregulation of PTN in ESCs from RIF and RPL patients (**Figures 3D,E**). Taken together, these findings indicate that PTN is predominantly expressed in ESCs and is markedly downregulated in pathological pregnancy conditions, including RIF and RPL.

### 3.4 PTN regulates endometrial stromal cell decidualization through the IGF-2 signaling axis

**Figure 4:**
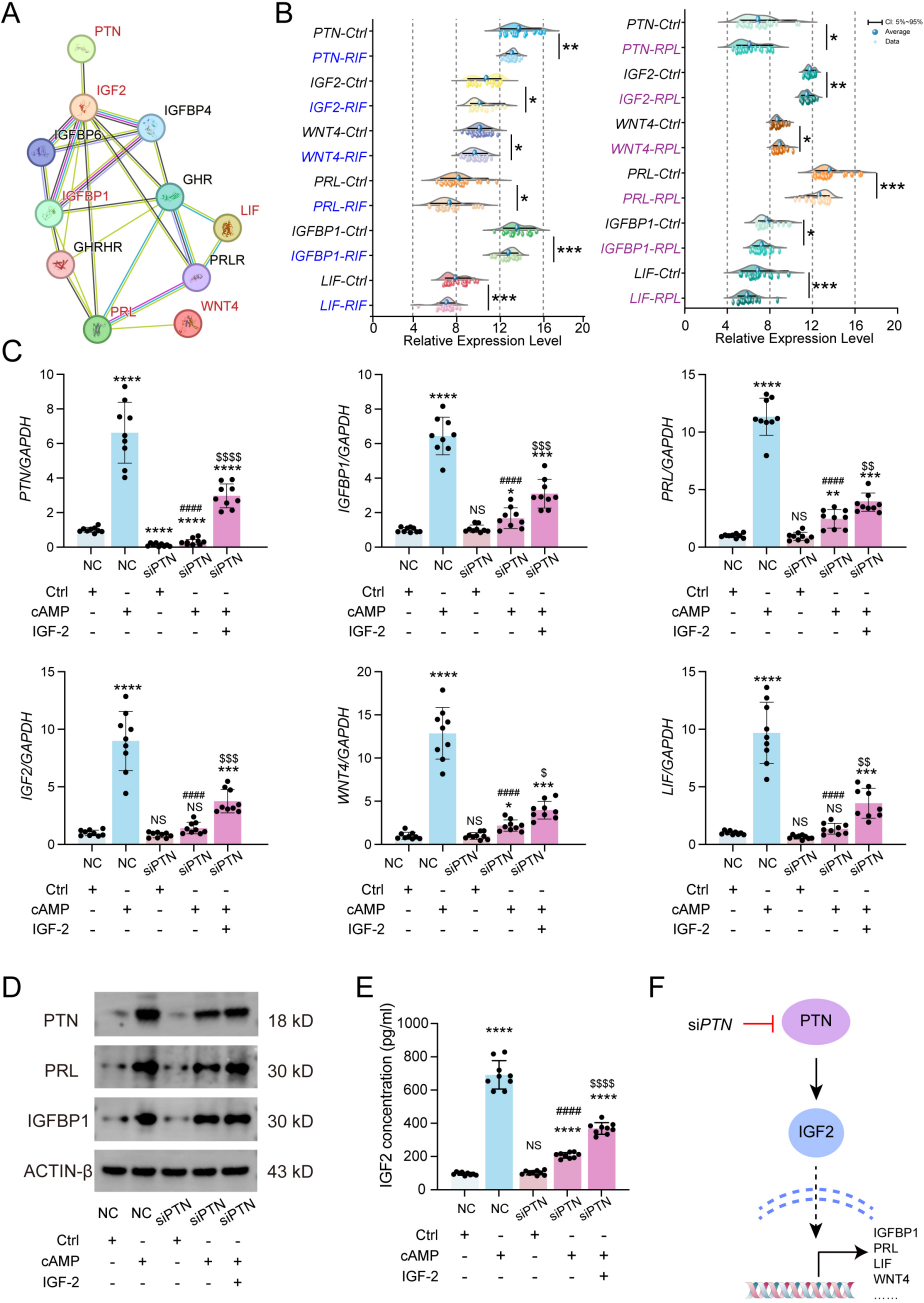
PTN regulates endometrial stromal cell decidualization through the IGF-2 signaling axis. **(A)** PPI network of PTN, IGF-2, IGFBP1, LIF, PRL, and other decidualization-related genes. **(B)** The expression levels of PTN and its interacting partners (IGF-2, WNT4, PRL, IGFBP1, and LIF) in the RIF (left) and RPL (right) datasets. **(C)** Expressions of decidualization-related genes (including *IGFBP1*, *LIF*, *PRL*, and *WNT4*) in NC or si*PTN* hESCs after treatment with control vehicle, cAMP, and IGF-2 via RT-qPCR (n = 9). **(D)** Immunoblotting for PTN, IGFBP1, and PRL expression levels with control vehicle, cAMP, and IGF-2. **(E)** ELISA for IGF-2 levels with control vehicle, cAMP, and IGF-2. **(F)** Schematic diagram of the experiments performed using the different treatments of hESCs. Data are presented as mean ± SEM and analyzed using t-test or one-way ANOVA test. * compared with NC treated with control vehicle, # compared with si*PTN* treated with control vehicle, $ compared with si*PTN* treated with control cAMP (NS, no significant difference; *p < 0.05, **p < 0.01, ***p < 0.001, ****p < 0.0001, ####p < 0.0001, $p < 0.05, $$p < 0.01, $$$p < 0.001, $$$$p< 0.0001). PPI, protein–protein interaction; RIF, recurrent implantation failure; RPL, recurrent pregnancy loss; hESCs, human endometrial stromal cells; cAMP, cyclic adenosine monophosphate.

To further explore the functional role of PTN in ESC decidualization, we constructed a PPI network based on PTN. The analysis revealed that PTN interacts with multiple decidualization-associated proteins (such as IGFBP1, PRL, WNT4, and LIF) through the intermediate factor IGF-2, suggesting that PTN may participate in the regulation of endometrial decidualization through IGF-2 (**Figure 4A**). We next evaluated the expression patterns of PTN and its interacting decidualization-related genes in RIF and RPL transcriptomic datasets. As shown in **Figure 4B**, PTN, together with IGF-2, IGFBP1, PRL, WNT4, and LIF, was significantly downregulated in both RIF and RPL compared with controls, indicating that dysregulation of this gene network is a common molecular feature of pathological pregnancy. Given the close association between PTN and IGF-2, we hypothesized that IGF-2 acts as a downstream effector of PTN in ESCs. To test this hypothesis, PTN was silenced in ESCs to mimic the pathological pregnancy state, and decidualization was induced using cAMP. It was demonstrated via qPCR analysis that cAMP treatment markedly upregulated the mRNA levels of decidualization markers (*IGFBP1*, *PRL*, *IGF-2*, *WNT4*, and *LIF*), whereas PTN knockdown significantly impaired these effects. Importantly, supplementation with exogenous IGF-2 partially restored the expression of decidualization-associated genes suppressed by PTN silencing (**Figure 4C**). Consistent with the transcriptional findings, Western blotting analysis (**Figure 4D**) and ELISA (**Figure 4E**) further confirmed that PTN knockdown reduced the protein levels of decidualization markers, including PRL and IGFBP1, while IGF-2 treatment rescued their expression. Collectively, these results demonstrate that PTN positively regulates ESC decidualization by modulating IGF-2 signaling. Disruption of the PTN/IGF-2 axis leads to impaired decidualization, providing a mechanistic link between PTN downregulation and the pathological features observed in RIF and RPL (**Figure 4F**).

### 3.5 IGF-2 promotes decidualization and rescues decidualization defects caused by PTN deficiency

**Figure 5:**
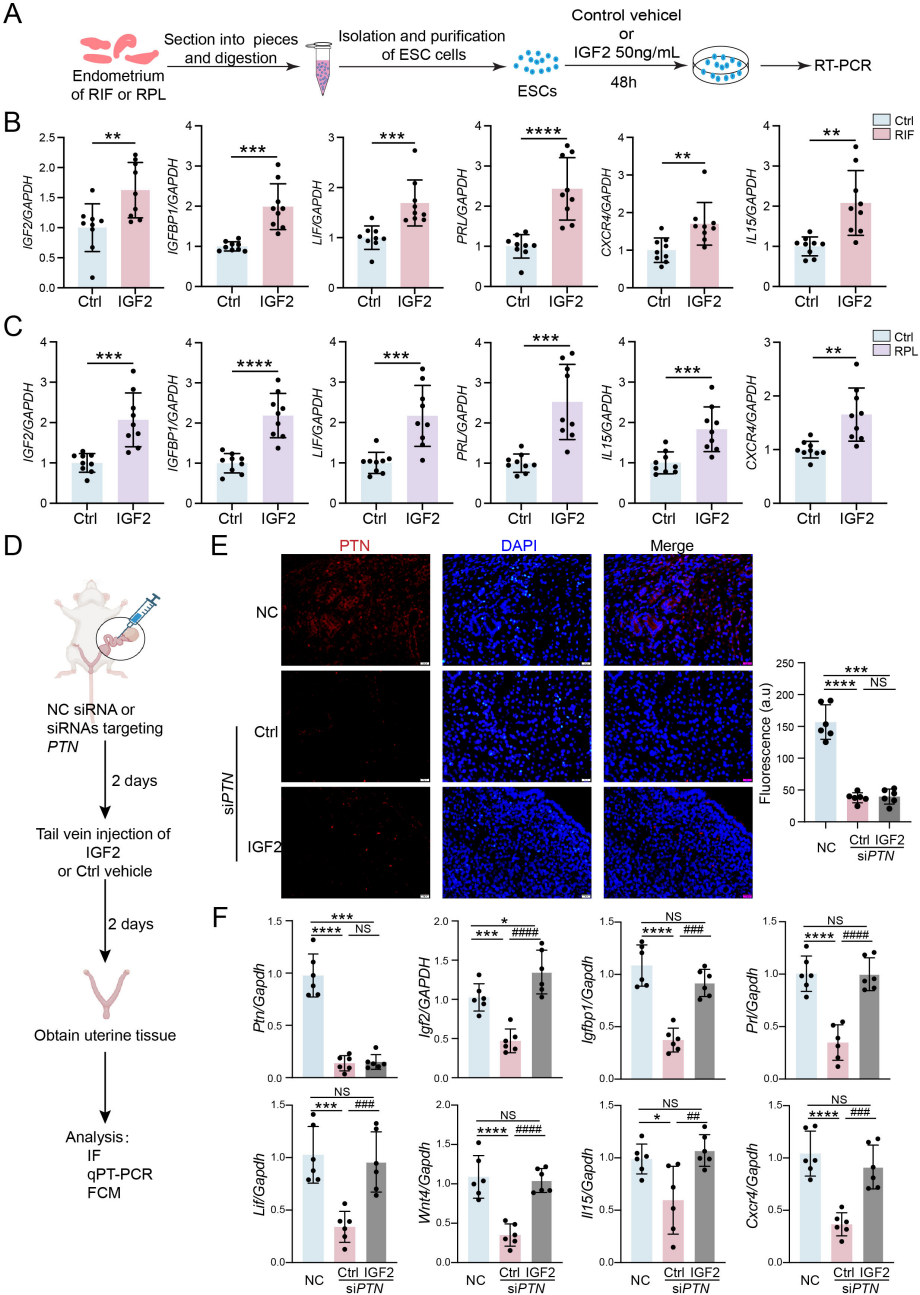
IGF-2 promotes decidualization and rescues decidualization defects caused by PTN deficiency. **(A)** Schematic diagram of the experiments performed using the different treatments of ESCs isolated from endometrium of RIF and RPL patients. **(B, C)** RT-qPCR analysis of levels of decidualization-related genes in ESCs isolated from endometrium of RIF **(B)** and RPL **(C)** patients after IGF-2 (50 ng/mL) treatment for 48 h (n = 9). **(D)** The flowchart depicts the steps involved in establishing a mouse model of intrauterine perfusion with siRNA-mediated knockdown of PTN and then supplemented with IGF-2 (50 ng/mL) through tail vein injection. **(E)** Expression of PTN after *Ptn* knockdown and supplemented with control vehicle or IGF-2 (50 ng/mL) in the mouse endometrium, measured by immunofluorescence, and quantitative analysis of immunofluorescence was performed (n = 6). **(F)** RT-qPCR analysis of levels of decidualization-related genes after *Ptn* knockdown and supplemented with control vehicle or IGF-2 (50 ng/mL) in the mouse endometrium (n = 6). Data are presented as mean ± SEM and analyzed using t-test or one-way ANOVA test. * compared with NC treated with control vehicle, # compared with si*PTN* treated with control vehicle (NS, no significant difference; *p < 0.05, **p < 0.01, ***p < 0.001, ****p< 0.0001, ## p < 0.01, ### p < 0.001, #### p < 0.0001). ESCs, endometrial stromal cells; PPI, protein–protein interaction; RIF, recurrent implantation failure.

To determine whether IGF-2 is involved in PTN-mediated regulation of endometrial decidualization, ESCs were isolated from patients with RIF and RPL and treated with exogenous IGF-2 *in vitro* (**Figure 5A**). The results showed that IGF-2 supplementation significantly upregulated the expression of multiple decidualization-related markers in ESCs derived from both RIF and RPL patients (**Figures 5B, C**). Furthermore, a mouse model with *in situ* knockdown of PTN in the uterus was established, followed by exogenous IGF-2 administration (**Figure 5D**). Immunofluorescence staining confirmed the efficient knockdown of PTN in the uterine endometrium (**Figure 5E**). Compared with the control group, IGF-2 treatment markedly enhanced the degree of endometrial decidualization in PTN-deficient mice (**Figure 5F**). Collectively, these results indicate that exogenous IGF-2 not only promotes decidualization in ESCs derived from RIF and RPL patients but also partially reverses the decidualization defects caused by PTN loss *in vivo*. These findings suggest that IGF-2 acts as a key downstream paracrine factor of PTN and plays an important compensatory role in the regulation of endometrial decidualization.

### 3.6 CD16^+^ NK cells and CD8^+^ T cells were elevated in RIF and RPL

**Figure 6:**
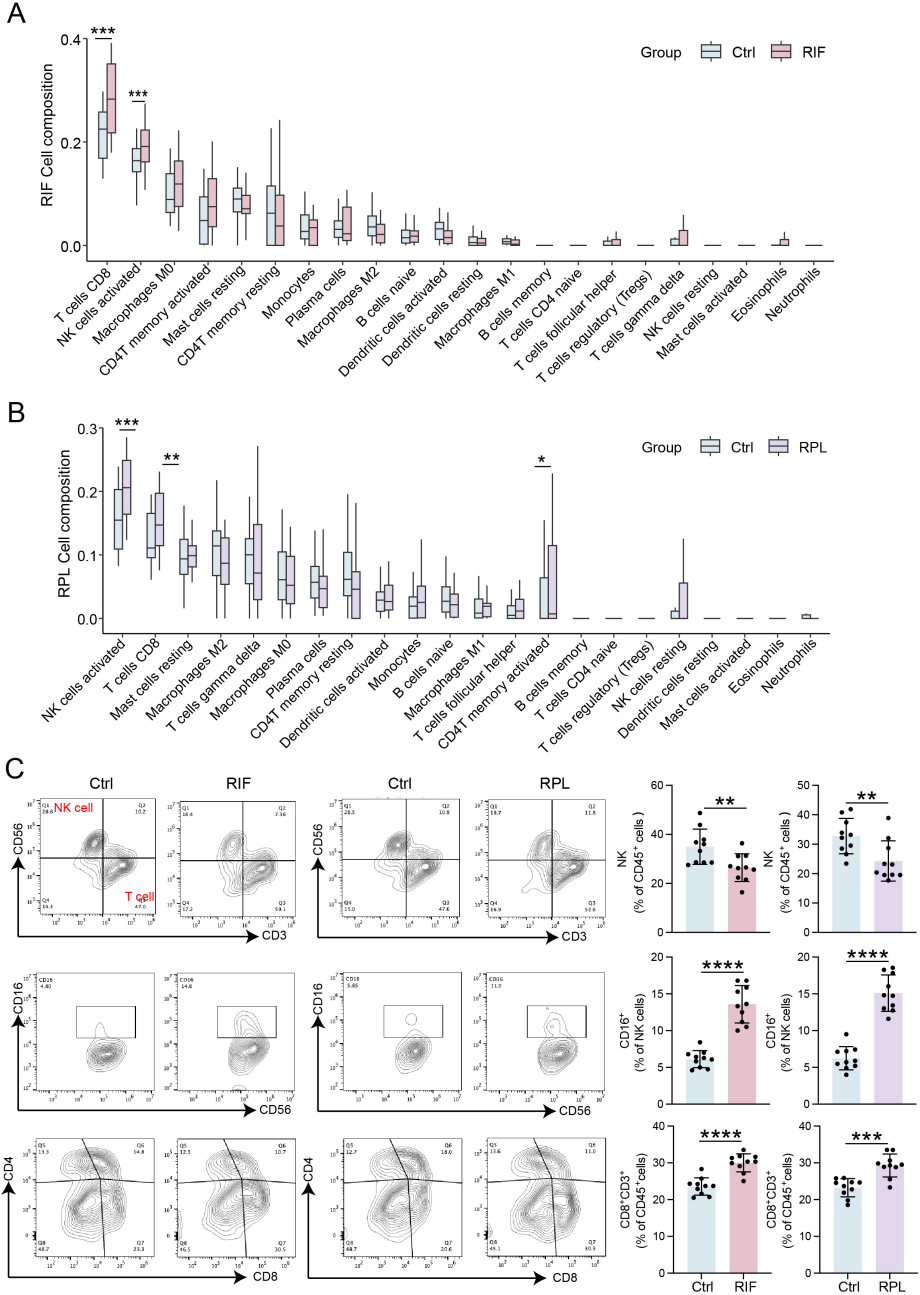
CD16^+^ NK cells and CD8^+^ T cells were elevated in RIF and RPL. **(A, B)** The difference in immune cell infiltration in RIF and RPL, respectively. **(C)** Flow cytometry analysis of proportions of CD16^+^ NK cells and CD8^+^ T cells in the endometrium of RIF and RPL patients (n = 10). Data are presented as mean ± SEM and analyzed using t-test (*p < 0.05, **p < 0.01, ***p < 0.001, ****p < 0.0001). RIF, recurrent implantation failure; PPI, protein–protein interaction.

Since RIF and RPL were both immune-related diseases, which was also confirmed in this study (**Figures 1D, E**), immune cell infiltration analysis was performed to clarify the immune regulation of RIF and RPL. Regarding the RIF patients and healthy controls, the proportion of 22 kinds of immune cells is shown in **Figures 6A**, in which CD8^+^ T cells and activated NK cells accounted for the highest proportion in RIF. A similar phenomenon was observed in RPL, in which CD8^+^ T cells and NK cells accounted for the highest proportion (**Figure 6B**). Consequently, the levels of NK and T cells were verified in RIF and RPL patients, respectively, via flow cytometry, and we found that the proportions of activated NK cells (CD16^+^ NK cells) and CD8^+^ T cells were elevated in RIF and RPL, respectively, which was in accordance with the previous immune infiltration analysis (**Figure 6C**).

### 3.7 PTN deficiency induces endometrial immune imbalance and leads to adverse pregnancy outcomes, which can be partially reversed by IGF-2

**Figure 7:**
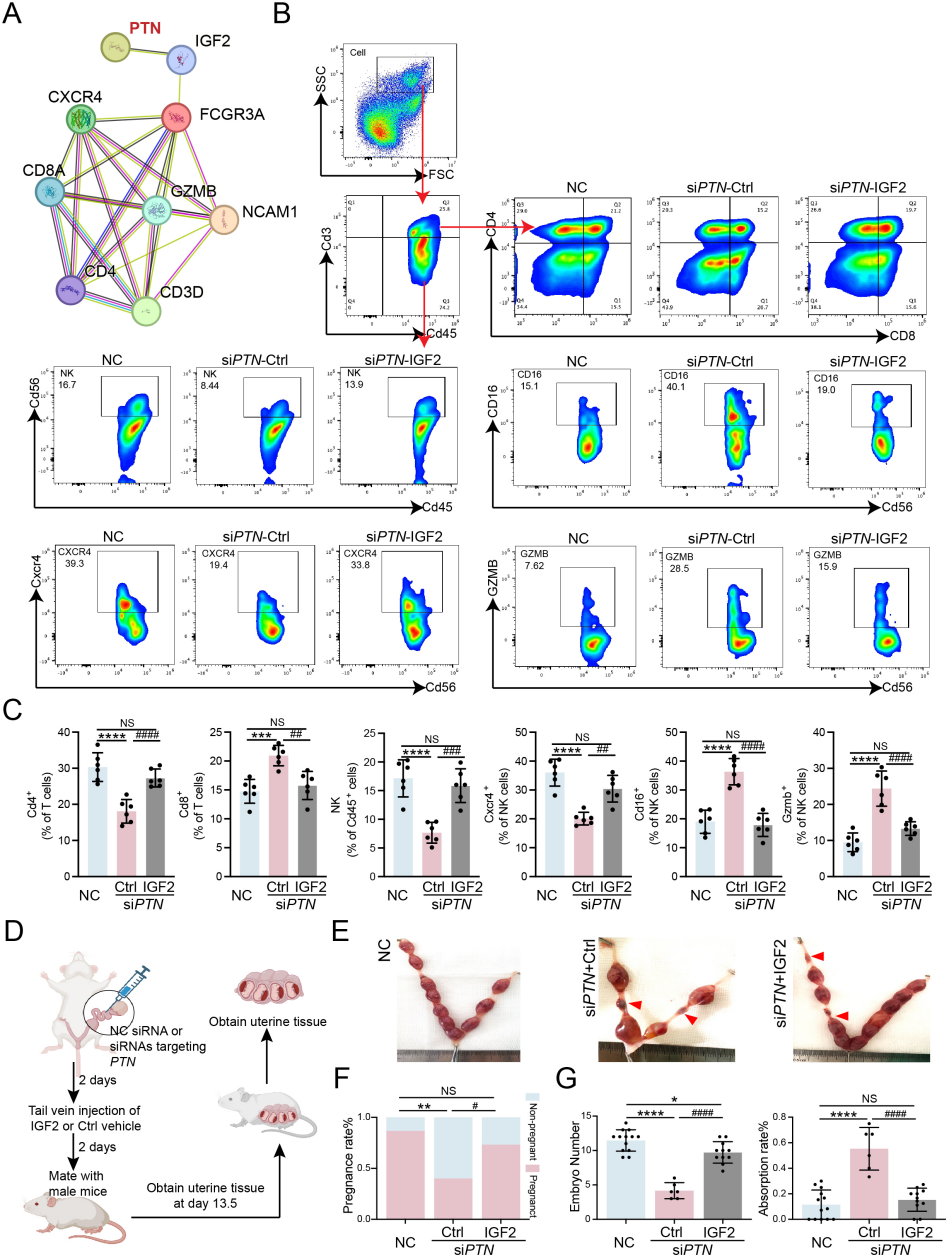
PTN deficiency induces endometrial immune imbalance and leads to adverse pregnancy outcomes, which can be partially reversed by IGF-2. **(A)** PPI network of PTN, IGF-2, CXCR4, CD4, CD8, CD56, and other immunoregulators. **(B, C)** Flow cytometry analysis **(B)** and statistical quantification **(C)** of cell populations of NK cells and T cells, and the expression levels of CD16, CXCR4, and GZMB in NK cell form the endometrial tissues of NC and si*PTN* mice treated with control vehicle or IGF-2 (50 ng/mL) (n = 6). **(D)** The flowchart depicts the steps involved in establishing a mouse model of intrauterine perfusion with siRNA-mediated knockdown of *Ptn* and then supplemented with IGF-2 (50 ng/mL) through tail vein injection for 2 days; then, these female mice were mated with fertile male mice; analysis of fertility, including pregnancy rate, IF, and fluorescence-activated cell sorting (FACS). were performed at gestational day 13.5. **(E)** Representative images of uteri from pregnant mice in the NC and si*PTN* mice treated with control vehicle or IGF-2 (50 ng/mL) at gestational day 13.5 (n = 6) (arrow shows the absorption site). **(F)** Pregnancy rate (%) in NC and si*PTN* mice treated with control vehicle or IGF-2 (50 ng/mL) (n = 15). **(G)** Quantification of embryo numbers (left) and absorption rate (right) per mouse in NC (n = 13) and si*PTN* mice treated with control vehicle (n = 6) or IGF-2 (50 ng/mL) (n = 11) at gestational day 13.5. Data are presented as mean ± SEM and analyzed using one-way ANOVA test or χ^2^ test. * Compared with NC treated with control vehicle, # compared with si*PTN* treated with control vehicle (NS, no significant difference; *p < 0.05, **p < 0.01, ***p < 0.001, ****p < 0.0001, ## p < 0.01, ### p < 0.001, #### p < 0.0001). IF, immunofluorescence.

We further performed PPI analysis of proteins associated with the PTN/IGF-2 axis and identified several immune-related proteins, including CD4, CD8A, CD3D, and GZMB (**Figure 7A**). Given that both RIF and RPL patients exhibited immune imbalance within the endometrial microenvironment, as shown in **Figure 6**, subsequently, immune cell proportions in the mouse model were analyzed, and the flow cytometry gating strategy is shown in **Figure 7B**. Quantitative analysis revealed that PTN knockdown recapitulated the immune microenvironment observed in the endometrium of RIF and RPL patients, characterized by a reduction in CD4^+^ T cells and an increase in CD8^+^ T cells and NK cells. Moreover, the elevated proportions of CD16^+^ NK cells and GZMB^+^ NK cells indicated enhanced NK cell cytotoxic activity. These results suggest that PTN deficiency induces a breakdown of immune tolerance in the endometrial microenvironment, which can be partially reversed by IGF-2 supplementation (**Figure 7C**). In addition to alterations in immune cell composition, PTN knockdown also led to adverse pregnancy outcomes in mice. Mice with uterine PTN knockdown exhibited a significantly reduced pregnancy rate, decreased embryo numbers, and an increased embryo resorption rate. Notably, tail vein injection of IGF-2 partially rescued these unfavorable pregnancy outcomes (**Figures 7D–F**). Collectively, these findings demonstrate that PTN deficiency disrupts endometrial immune homeostasis, leading to immune overactivation and impaired pregnancy outcomes, while IGF-2 supplementation can partially restore immune balance and improve reproductive outcomes, further supporting a critical functional role of the PTN/IGF-2 axis in maintaining endometrial immune tolerance and successful pregnancy.

## 4 Discussion

In this study, we explored the common molecular basis of RIF and RPL using an integrative co-disease analysis strategy and identified PTN as a key hub gene shared by both conditions. Subsequent functional analyses revealed that PTN plays an essential role in regulating endometrial function during early pregnancy. Loss of PTN impaired decidualization and disturbed immune homeostasis in the endometrial microenvironment, leading to compromised fertility and adverse pregnancy outcomes in mice. Importantly, these defects were partially ameliorated by exogenous IGF-2, suggesting that PTN exerts its effects, at least in part, through an IGF-2-mediated paracrine mechanism.

The PTN gene is located on chromosome 6 in mice and chromosome 7 in humans (22). The protein encoded by PTN, pleiotrophin, is a fibroblast mitogenic protein. First isolated in 1989–1990 from various tissues, including bovine uterus, neonatal rat brains, and mouse bone tissue, PTN belongs to the secreted heparin-binding cytokine family, which is known for its high affinity for heparin. PTN has been extensively studied for its roles in cell proliferation, angiogenesis, and tissue remodeling (23–25). While its functions have been extensively studied in the context of neurodevelopment and tumor biology, its role in reproductive physiology remains largely unexplored. To our knowledge, this is the first study to demonstrate the functional involvement of PTN in fertility regulation, suggesting that PTN may participate in the regulation of embryo implantation and pregnancy maintenance and highlighting its potential contribution to the pathogenesis of both RIF and RPL.

Recent research has highlighted the growing interest in PTN for its role in immune regulation. Studies have demonstrated that PTN influences immune responses through multiple mechanisms. For instance, PTN has been shown to regulate neuroinflammation by enhancing lipopolysaccharide (LPS)-induced microglial activation, suggesting that PTN may modulate immune responses in the central nervous system (CNS) by affecting microglial activity (26). Additionally, PTN plays a role in the recovery phase of autoimmune CNS diseases. Research has indicated that astrocyte-derived PTN contributes to the remission stage of experimental autoimmune encephalomyelitis (EAE), helping to limit chronic CNS inflammation (27). In cancer research, PTN has been identified as a promoter of breast cancer metastasis and is associated with poor prognosis. It may contribute to the formation of a pro-metastatic immune niche by modulating immune cells within the tumor microenvironment (28). Overall, PTN exhibits multifaceted immunoregulatory functions spanning neuroinflammation, autoimmune diseases, and tumor immunology. Given the complex interplay of immune tolerance, endometrial remodeling, and vascularization required for successful implantation and pregnancy, aberrant PTN expression may disrupt these processes, contributing to the pathogenesis of both RIF and RPL.

Decidualization of ESCs is a hormonally driven differentiation process that transforms the proliferative endometrium into a receptive and supportive decidua necessary for embryo implantation and pregnancy progression. Impaired decidualization is increasingly recognized as a key pathological feature in reproductive failures such as RIF and RPL (e.g., deficiencies in decidual cell transformation are associated with implantation and early gestational failures in affected women) (29). In the present study, we show that PTN deficiency significantly attenuates decidual marker expression in ESCs and disrupts *in vivo* decidual responses, indicating that PTN may function as a positive regulator of stromal cell decidualization. Mechanistically, PTN is a secreted growth factor known to modulate cell differentiation and tissue remodeling in other contexts, and our observation that exogenous IGF−2 partially restores decidualization in PTN−deficient ESCs supports a paracrine component to this regulatory axis. Components of the IGF signaling network, including IGF−2, have also been observed to be upregulated during decidualization in human ESC models, further implicating IGF family signaling in stromal differentiation processes relevant to pregnancy establishment (30). In addition, PTN-mediated inhibition of receptor protein tyrosine phosphatase beta/zeta (RPTPβ/ζ) leads to sustained activation of substrates such as β-catenin, thereby influencing Wnt signaling, which is essential for endometrial epithelial remodeling and receptivity (31, 32). Clinically, lower PTN expression in the endometrium of RIF and RPL patients may contribute to defective decidualization, compromising the establishment of a receptive endometrial environment and increasing the risk of implantation failure and pregnancy loss.

In addition to regulating stromal cell decidualization, PTN appears to orchestrate the local immune milieu, with deficiency resulting in an immune imbalance that may compromise pregnancy success. Maternal immune tolerance at the maternal–fetal interface is essential for successful embryo implantation and pregnancy maintenance. This complex immunological environment balances defense against pathogens with tolerance toward the semi−allogeneic conceptus, involving regulatory adaptations in decidual immune cells, cytokine networks, and trophoblast–immune interactions that promote a tolerogenic milieu (33). Recent studies have highlighted that specialized decidual immune populations, including regulatory T cells (Tregs) and tolerogenic uterine NK cell subsets, are required to maintain maternal–fetal tolerance, and disruption of these populations is associated with pregnancy loss in both clinical and preclinical models (34). Furthermore, recent evidence suggests that PTN contributes to immune regulation by promoting M2 macrophage polarization and modulating dendritic cell and T-cell activity, thereby creating an immune-tolerant microenvironment necessary for embryo survival (27, 35). In our study, PTN deficiency induced an immune−imbalanced endometrial microenvironment characterized by increased cytotoxic immune cell proportions, consistent with a breakdown of immune tolerance and adverse reproductive outcomes. Conversely, IGF−2 supplementation partially restored immune balance, suggesting that the PTN/IGF-2 axis may support the establishment of a tolerogenic decidual immune niche. These findings align with emerging evidence that immune dysregulation at the maternal–fetal interface contributes to recurrent pregnancy loss and implantation failure, underscoring the importance of coordinated stromal–immune crosstalk for reproductive success (36). Collectively, this evidence suggests that PTN may act as a multi-functional regulator at the intersection of angiogenesis, immune tolerance, and cellular remodeling, making it highly relevant to the pathophysiology of RIF and RPL.

Although our flow cytometry analysis provided an overview of immune alterations associated with PTN expression, the current study primarily focused on major immune subsets, particularly NK cells and CD8^+^ T cells, based on their prominent changes and well-established roles in implantation and early pregnancy. Accumulating evidence indicates that uterine NK (uNK) cells are critical for spiral artery remodeling, trophoblast invasion, and placental development (37, 38), while CD8^+^ T cells participate in immune tolerance and tissue homeostasis at the maternal–fetal interface (39, 40). Nevertheless, other immune populations, including Treg and myeloid-derived suppressor cell (MDSC) subtypes, also play indispensable roles in maintaining immune balance, promoting decidualization, and supporting embryo implantation (41, 42). However, functional immune tolerance, such as cytokine secretion patterns and immune cell–cell interactions, was not systematically investigated in the present study. These limitations highlight the need for more comprehensive immune profiling in future work, combining high-dimensional flow cytometry, single-cell or spatial transcriptomics, and functional assays, to fully delineate the immune regulatory network orchestrated by the PTN/IGF-2 axis during endometrial remodeling, implantation, and pregnancy maintenance.

Although the precise molecular mechanisms by which PTN regulates IGF-2 remain unclear, PTN is known to signal through multiple cell surface receptors, including anaplastic lymphoma kinase (ALK) and RPTPβ/ζ, which can activate downstream pathways such as PI3K–AKT, MAPK, and JAK–STAT (43, 44). Binding of PTN to these receptors triggers receptor autophosphorylation or inactivation, leading to downstream signaling that promotes cell survival, proliferation, and angiogenesis (45). PTN may also activate the MAPK/ERK pathway, facilitating trophoblast migration and invasion, a process reminiscent of tumor cell metastasis (46). These processes are particularly relevant in the context of endometrial stromal cell decidualization and vascular remodeling, both of which are essential for embryo implantation and early pregnancy maintenance (45, 47). Moreover, IGF-2 functions as a key downstream effector of PTN in endometrial cells. Mechanistically, IGF-2 may mediate the effects of PTN through the PI3K/AKT and MAPK (ERK1/2 and p38) pathways (48, 49). Nevertheless, the direct molecular linkage between PTN receptor engagement and IGF-2 transcriptional or post-transcriptional regulation remains to be elucidated, and further mechanistic studies are required to delineate this regulatory cascade in detail.

From a clinical perspective, PTN holds promise as a novel biomarker for identifying patients at risk for RIF and RPL. Furthermore, targeting the PTN/IGF-2 axis may offer new therapeutic strategies to improve reproductive outcomes. However, several limitations should be acknowledged. While our study included both human *in vitro* and murine *in vivo* models, certain species-specific differences may influence the generalizability of our findings. Although the core PTN/IGF-2 signaling mechanism appears conserved, further validation in larger and more diverse human cohorts, as well as in different endometrial cell types, will be important to confirm the universality and clinical applicability of these findings. Nevertheless, integrated transcriptomic or spatial profiling may also help clarify regulatory nuances and strengthen the translational relevance of PTN-centered interventions within the endometrial microenvironment.

In conclusion, our study identified PTN as a central regulator of endometrial function, coordinating stromal cell decidualization and a tolerogenic immune microenvironment. Dysregulation of the PTN/IGF-2 axis may contribute to pathological pregnancies, including RIF and RPL, and represents a potential target for future translational interventions aimed at improving pregnancy outcomes.

## Data Availability

The original contributions presented in the study are included in the article/**Supplementary Material**. Further inquiries can be directed to the corresponding authors.

## Supplementary material

Supplementary Figure 1 Heatmaps and PCA of top 50 DEGs in RIF and RPL. **(A)** Expression of top 50 genes in RIF and Ctrl groups was presented by heatmap. **(B)** PCA analysis of RIF datasets GSE111974, and GSE188409 expression profiles before (up) and after (down) normalization. **(C)** Expression of top 50 genes in RPL and Ctrl groups was presented by heatmap. **(D)** PCA analysis of RPL datasets GSE26787, and GSE165004 expression profiles before (up) and after (down) normalization.

Supplementary Figure 2 PPI network analysis of Co-Expressed DEGs in RIF and RPL. **(A)** The top 10 hub genes of 10 patterns were discovered by Cytoscape software (version 3.7.2, cytoHubba plug-ins). **(B)** The frequency of hub genes.

Supplementary Figure 3 Validation of PTN know down by siRNA in hESC cell line by IF. PTN was knocked down in the hESC cell line, with a non-targeting control group set in parallel. Cellular immunofluorescence (IF) staining was performed to verify PTN expression. A significant reduction in PTN fluorescence intensity in the PTN knockout group, compared with the control group, will confirm the specificity of the PTN antibody. All supplementary experiments were performed in triplicate to ensure reproducibility. (Blue: DAPI; Green: Vimentin; Red: PTN).
