# Supplementary figures and images for: PTN/IGF-2 signaling modulates endometrial decidualization and immune cell trafficking to facilitate pregnancy maintenance
Source: Front Immunol. 2026 Mar 23;17:1790942. doi: 10.3389/fimmu.2026.1790942 (PMC13050712; doi:10.3389/fimmu.2026.1790942)

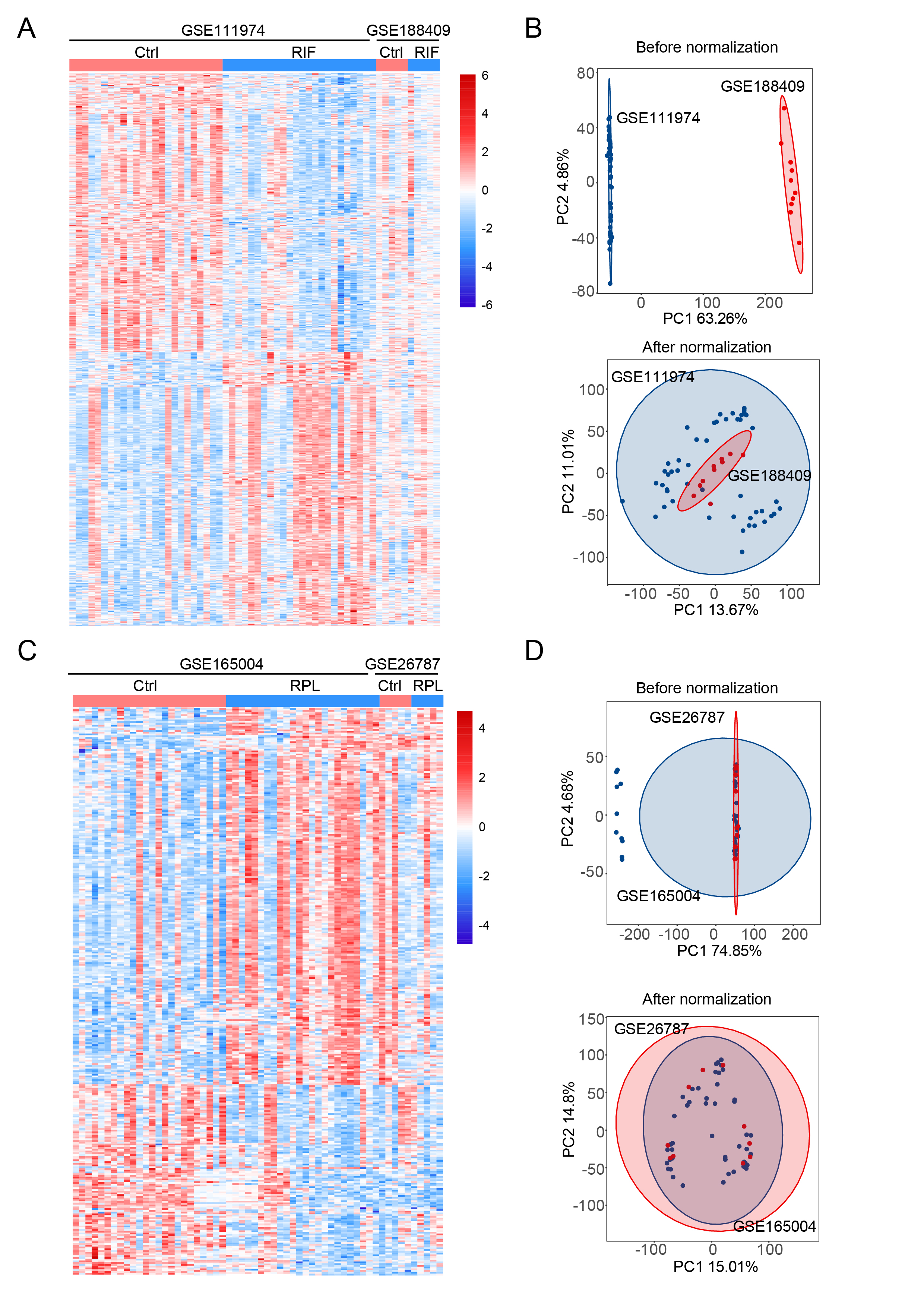

Supplement: Supplementary Figure 1 — Heatmaps and PCA of top 50 DEGs in RIF and RPL. (A) Expression of top 50 genes in RIF and Ctrl groups was presented by heatmap. (B) PCA analysis of RIF datasets GSE111974, and GSE188409 expression profiles before (up) and after (down) normalization. (C) Expression of top 50 genes in RPL and Ctrl groups was presented by heatmap. (D) PCA analysis of RPL datasets GSE26787, and GSE165004 expression profiles before (up) and after (down) normalization. [file Image1.jpeg]

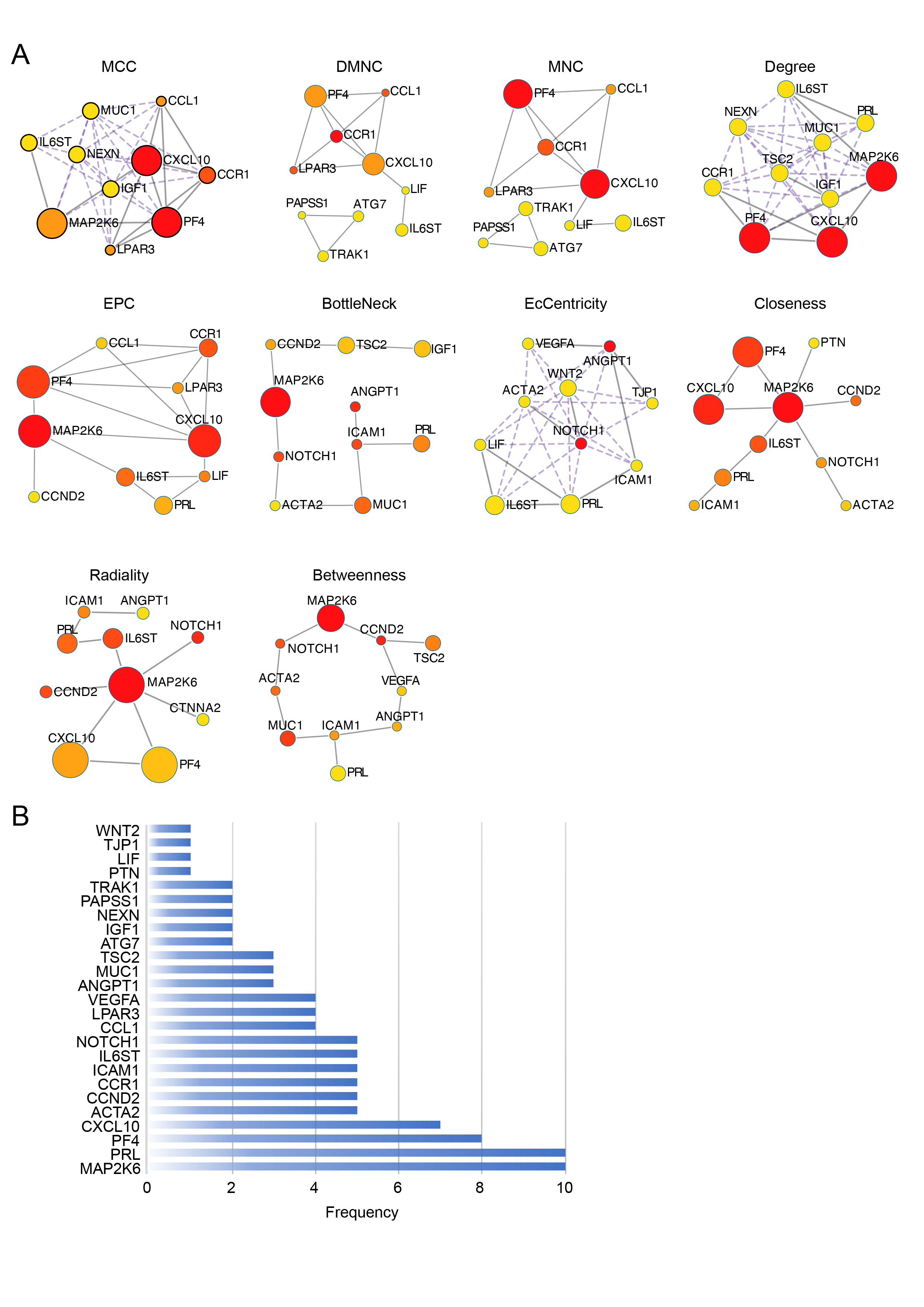

Supplement: Supplementary Figure 2 — PPI network analysis of Co-Expressed DEGs in RIF and RPL. (A) The top 10 hub genes of 10 patterns were discovered by Cytoscape software (version 3.7.2, cytoHubba plug-ins). (B) The frequency of hub genes. [file Image2.jpeg]

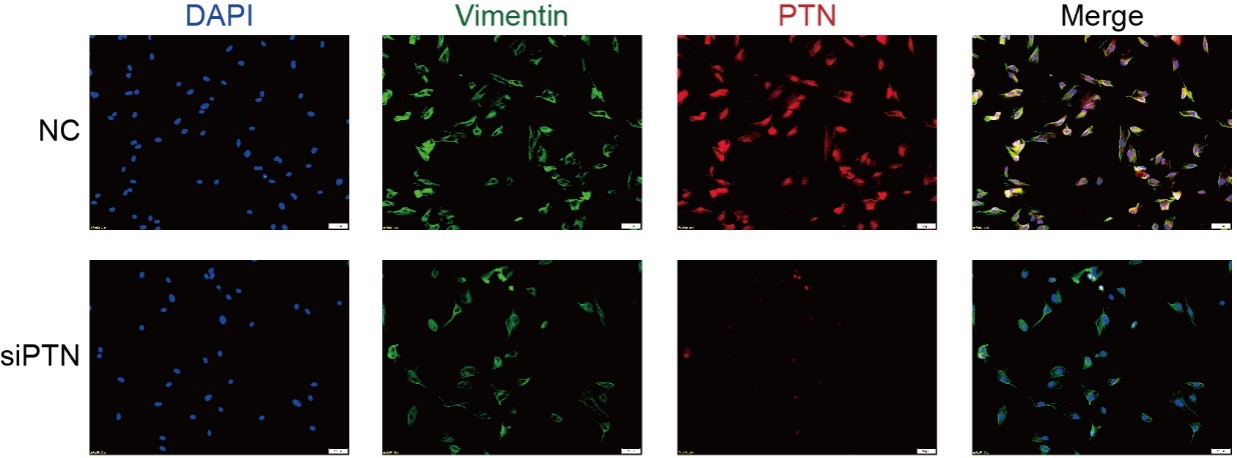

Supplement: Supplementary Figure 3 — Validation of PTN know down by siRNA in hESC cell line by IF. PTN was knocked down in the hESC cell line, with a non-targeting control group set in parallel. Cellular immunofluorescence (IF) staining was performed to verify PTN expression. A significant reduction in PTN fluorescence intensity in the PTN knockout group, compared with the control group, will confirm the specificity of the PTN antibody. All supplementary experiments were performed in triplicate to ensure reproducibility. (Blue: DAPI; Green: Vimentin; Red: PTN). [file Image3.jpeg]
